# Clinical and genomic profiling of *Klebsiella pneumoniae* in liver abscesses

**DOI:** 10.1080/21505594.2026.2670805

**Published:** 2026-05-12

**Authors:** Felix Aurnhammer, Maxim M. Baltzer, Jamil Butt, Lisa S. Marr, Anca Rath, Baerbel Kieninger, Wulf Schneider-Brachert, Christian Utpatel, Stefan Niemann, Eike Steinmann, Alexander Dechêne, Joerg Steinmann, Bernd Neumann

**Affiliations:** a Institute of Clinical Microbiology, Infectious Diseases and Infection Control, Paracelsus Medical University, Nuremberg General Hospital, Nuremberg, Germany; b Department of Infection Control and Infectious Diseases, University Hospital Regensburg, Regensburg, Germany; c Institute of Microbiology and Hygiene, University Regensburg, Regensburg, Germany; d Research Center Borstel, Leibniz Lung Center, Molecular and Experimental Mycobacteriology, Germany; e German Centre for Infection Research (DZIF), Partner Site Hamburg-Lübeck-Borstel-Riems, Borstel, Germany; f EPHE, PSL University, Paris; Institut de Systématique, Évolution, Biodiversité (ISYEB), Muséum national d’Histoire naturelle, CNRS, Sorbonne Université, EPHE, Université des Antilles, Paris, France; g Department of Molecular and Medical Virology, Ruhr University Bochum, Bochum, Germany; German Centre for Infection Research (DZIF), External Partner Site, Bochum, Germany; h Department of Gastroenterology, Hepatology and Endocrinology, Paracelsus Medical University, Nuremberg General Hospital, Nuremberg, Germany

**Keywords:** Virulence genes, hypervirulence, surveillance, infection prevention, liver abscess, diabetes

## Abstract

*Klebsiella pneumoniae* (*K. pneumoniae*) is an opportunistic Gram-negative pathogen responsible for a variety of infections, including liver abscesses (LA). These are associated with hypervirulent *K. pneumoniae* (hvKp). This study aimed to characterize the clinical and molecular features of *K. pneumoniae* from patients with LA at a German tertiary care hospital over a 2-y period (2022–2023). Among 111 recorded LA, *K. pneumoniae* was detected in 27% (*n* = 30) of cases. Of these, 12 patients had monomicrobial LA. Clinical metadata, including patient demographics, underlying diseases, and microbiological findings, were analyzed and correlated with genomic data to explore associations between genetic traits and clinical presentations. We identified seven cases of hypervirulent *K. pneumoniae* liver abscesses (hvKpLA) and five cases of non-hypervirulent *K. pneumoniae* liver abscesses (KpLA). The study found that hvKp was most commonly associated with specific sequence types (ST23-KL1, ST86-KL2) and hypervirulence-associated plasmids. Phylogenetic analysis revealed high genetic diversity, with no evidence of nosocomial transmission. Notably, diabetes mellitus (DM) was observed for five cases and may represent a risk factor for severe *K. pneumoniae* infections. In conclusion, monomicrobial *K. pneumoniae* LA infections were caused by hypervirulent strains in 58% of cases. Further, an hvKp case prevalence of 6.3% was estimated among all recorded liver abscesses. These findings contribute to a better understanding of the molecular epidemiology of hvKp and emphasize the importance of integrating clinical and genomic data for more accurate risk stratification and surveillance.

## Introduction

*Klebsiella pneumoniae* (*K. pneumoniae*) is an opportunistic Gram-negative pathogen known for the worldwide spread of clonal genetic lineages associated with various antibiotic resistances [1–3]. The “classic” *K. pneumoniae* (cKp) causes a range of infections, including sepsis and pneumonia, which particularly affect immunocompromised patients in hospitals and healthcare facilities [4–7]. In addition to the cKp pathotype, another variant has emerged, known as hypervirulent *K. pneumoniae* (hvKp) [8–10]. HvKp was first described in Asia in the 1980s and has since been identified and reported internationally [11–14]. The hvKp pathotype is characterized by pronounced clinical manifestations, which often lead to pyogenic liver abscesses (LA) and metastatic spread to multiple sites of infection [15].

Genome sequencing revealed the association of hypervirulence-mediating plasmids in LA case reports; with potential molecular marker genes as *rmpADC* (regulator of mucoid phenotype A), *rmpA2* (regulator of mucoid phenotype A2), *iutA* (receptor gene of the aerobactin operon), *clbB* (polyketide synthesis enzyme of colibactin operon), *ybt* (salicylate synthase of yersiniabactin operon), *peg-344* and *iroB* (glycosyltransferase of salmochelin) [15–20]. These genes are under investigation, since their contributions to the clinical manifestation have not been conclusively clarified. Despite their expanded virulence gene pool, most hvKp strains remain susceptible to antibiotic treatments. The spread of successful hvKp clones such as ST11, ST23, ST65, ST86 and ST380, which carry hypervirulence plasmids, has favored the dissemination of this pathotype within cKp-dominated populations adapted to hospital conditions [21]. Hospital outbreaks with hvKp have now been reported in several countries, including Germany, China, and Italy [22–24]. Most studies focus either on cases or molecular epidemiology. No systematic studies with data on case prevalence have been published in Western countries to date.

In previous studies, we identified putative hvKp strains from microbiological routine diagnostics upon patient admission [25,26]. It is noteworthy that infections with hvKp are predominantly acquired in outpatient settings and do not usually affect high-risk groups such as newborns or geriatric patients [15,27–29]. An increasing number of case reports on hvKp suggest a connection with patients living with diabetes mellitus (DM) [30,31]. A poorly controlled diabetic metabolism appears to contribute to a more severe course of the disease [32]. This led to the hypothesis that DM could be a risk factor for severe hvKp infections [32,33]. In addition, DM-patients with *K. pneumoniae* infections, but without LA, have also been reported [34]. The classic clinical presentation of LA with hypervirulent *K. pneumoniae* (hvKpLA) is increasingly being refuted by case reports documenting infections at various sites [27,29,35,36].

The aim of this study was to characterize cases of hvKp infections in a German tertiary care hospital (>2,200 beds) over a period of 2 y. We included all cases of monomicrobial liver abscesses from which *K. pneumoniae* was isolated. We compared the clinical presentations, preexisting conditions and microbiological characteristics. In addition, analyses based on whole-genome sequencing (WGS)-based were performed to thoroughly comprehensively the isolated *K. pneumoniae*. With this approach, the study aims to further fill the gaps in knowledge about hvKp liver abscesses.

## Methods

### Inclusion criteria, specimens and bacterial isolates

All LA from patients aged 18 y and older were recorded in 2022 and 2023. Microbiological diagnostics were realized for all cases, including identification of bacterial genera. The specimens obtained included blood, respiratory samples, biopsies, drainage, and puncture material. All available *K. pneumoniae* isolates from these patients were collected. To distinguish the monomicrobial status, cases were selected in which no second bacterial species were detected using culture methods. From 1 January 2022 to 31 December 2023, all cases with monomicrobial detection of *Klebsiella pneumoniae* were prospectively collected and included in this study. Two isolates (SPK0391-1 and SPK0391-2) were previously included in a surveillance study by Wahl et al. [37]. Species identification was performed using matrix-assisted laser desorption ionization time-of-flight (MALDI-TOF) mass spectrometry (Bruker Daltonik GmbH, Bremen, Germany). The string-test for hypermucoviscous phenotype was applied as described before [38]. Susceptibility testing of all isolates was executed according to EUCAST guidelines, utilizing the disc diffusion or semi-automated antimicrobial susceptibility testing using the Vitek 2 (bioMérieux, Marcy-l’Etoile, France) system.

### Patient metadata

The microbiological diagnostic results and basic patient metadata including age group and patient gender of all documented liver abscesses were compiled retrospectively. Eventual cases without microbiological diagnostics were included for statistics. For each included case with monomicrobial *K. pneumoniae*, further metadata were extracted retrospectively, including information on gender, age, underlying medical conditions, and travel history, where available. Relevant case-specific information was also documented, as applicable. All cases and patient metadata were completely anonymized. Categorial variables were presented as counts and percentages and were compared using the chi-square (χ^2^) tests or Fisher’s exact test, as appropriate depending on expected cell counts. All tests were two-sided, and *p*-values *p* < 0.05 were considered statistically significant. No adjustments or multiple testing were performed.

### Whole-genome sequencing and downstream analyses

All available *K. pneumoniae* isolates from the included patients underwent WGS. The DNA was extracted using the QIAmp DNA Mini Kit (#54304; Qiagen Diagnostics GmbH, Hilden, Germany) and quantified using a Qubit 4 fluorometer with the dsDNA HS Assay Kit (Thermo Fisher Scientific, Bremen, Germany). Library preparation was performed using Illumina Nextera reagents (Illumina GmbH, Berlin, Germany). WGS was performed on the Illumina NextSeq platform (Illumina GmbH, Berlin, Germany), generating 2 × 150 nucleotide paired-end reads. Raw read was quality checked using Falco (v1.2.4) [39].

*De novo* assembly of paired-end reads was performed using the SPAdes assembler (v3.15.4) integrated into the Galaxy (v23.2) web platform [40]. Quality control for assembled datasets was realized using BUSCO [41]. The reconstructed sequences were used for strain characterization and phylogenetic investigations. Species verification was realized with the Type Strain Genome Server (TYGS) [42]. To investigate antibiotic resistance genes and mobile genetic elements, the web tools of the Center for Genomic Epidemiology were used, including ResFinder (v4.1) and MobileElementFinder (v1.0) [43,44]. Screening for hypervirulence-associated genes was done using Kleborate (v3.2.3); and Geneious Prime (v2023.0.1) was used to validate the results on *rmpA*/*A2* by reference alignment (https://www.geneious.com/) [45]. Capsule typing was realized by using the Kaptive tool (v3.1.0) [46]. Multi-locus sequence typing (MLST), core-genome MLST (cgMLST), and core-SNP analysis for phylogenetic investigation were performed using the online platform Pathogenwatch [47]. According to the <10 allele difference threshold, isolate pairs falling below were evaluated as potential outbreak strains and further epidemiological patient records were investigated [48]. Phylogenetic trees were visualized using iTOL (v6.8.1) [49].

## Results

### Records of liver abscesses

**Figure 1.**
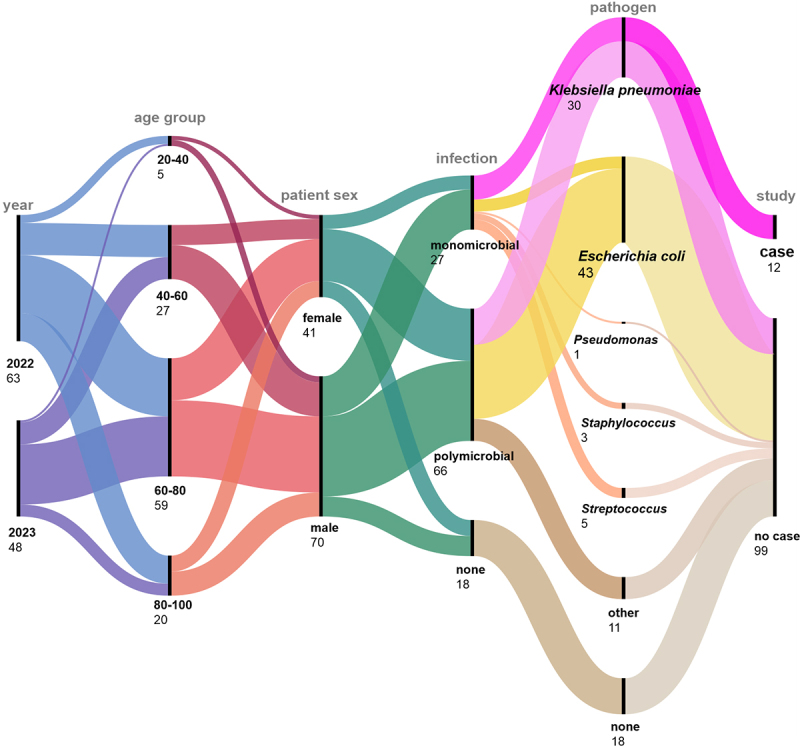
Presentation of all LA cases as alluvial diagram. The figure illustrates the year of case record (2022/2023), age groups (20-y intervals), patient sex (female, male), the results of microbiological investigations (monomicrobial, polymicrobial, none), pathogen detection, *K. pneumoniae* ‘cases’ for genomic investigations.

All patient records were reviewed to provide an overview of the general presentation of LA diagnoses. Over a period of 2 y, 111 patients were diagnosed with LA (Figure 1).

As shown in Figure 1, 56.8% of the cases occurred in 2022. Interestingly, significantly more male patients (63.1%) than female patients (χ^2^ testing; *p* = 0.00591) were recorded. In almost two-thirds of cases (59.5%), two or more bacteria were identified (see: Figure 1, polymicrobial). The highest percentage was attributable to *E. coli* (38.7%). *K. pneumoniae* was identified in 27% (30/111) of all patients, representing 16.2% of polymicrobial and 10.8% of monomicrobial LA. Monomicrobial *K. pneumoniae* accounted for 44.4% (12/27) of all recorded monomicrobial LA patients, which was twice as much as *E. coli* (6/27). The LA without microbiological identifications accounted for 16.2% (see: Figure 1, infection “none;” pathogen “none”). This group included also three cases where no microbiological investigation was performed.

The age of patients ranged from 21 to 93 y (mean: 65.81 y; median: 68 y; compare Figure 1, age group). The age range was similar for monomicrobial *K. pneumoniae* LA (mean: 67.38 y; median: 69 y). While the total number of LA was higher in 2022, the proportion of monomicrobial *K. pneumoniae* abscesses was higher in 2023 (*n* = 7; 14.6%) compared to 2022 (*n* = 5; 8%). For comparison, between 2022 and 2023, 6,108 *K. pneumoniae* isolates were collected from 3,456 patients during routine diagnostics. Therefore, isolates obtained from LA account for 0.4% of all clinical *K. pneumoniae.*

### WGS-based isolate characterization

**Figure 2.**
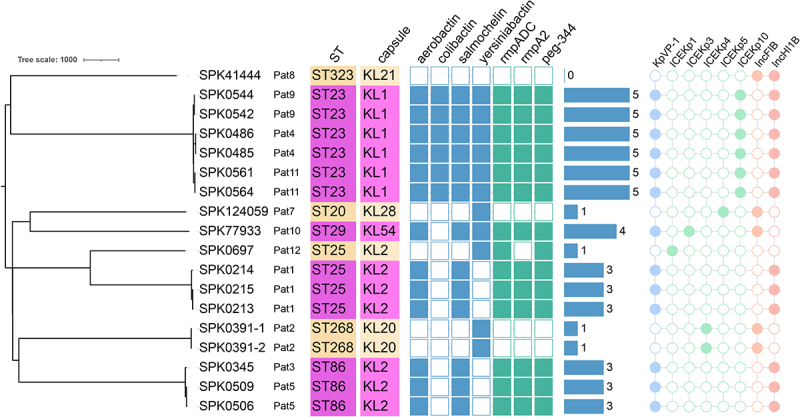
Results of genotyping and phylogenetic investigations. The results are based on analyses using the Pathogenwatch software suite, including coreSNP-based phylogeny. Multi-locus sequence typing (MLST) was used to determine sequence types (ST), and the Kleborate tool was employed to identify *Klebsiella* capsule types (KL) and virulence factors (detectable: filled square; undetectable: empty square). The virulence score (score range: 0-to-5; shown as bars) was determined by blue-colored virulence factors. In addition, identified mobile genetic elements and plasmids associated with virulence traits are visualized (detectable: filled circle; undetectable: empty circle).

Of the 12 cases to be considered for WGS-based investigations, 18 isolates were obtained from 11 patients (see Table 1). Specifically, two isolates from patients Pat2, Pat4, Pat5, Pat9, and Pat11, and three isolates from patients Pat1, were sequenced (Figure 2). Unfortunately, no isolate from Pat6 was available for sequencing.

**Table 1.** Patients with clinical presentations and anamnestic meta-data included in this study.

Patient	Sex	Year	Clinical presentation						Underlying condition and further anamnesis			Intervention	Microbiological diagnostic features		
			Abscess diameter^1^ [mm]	Material^2^	Documented BSI	Disseminated infection^3^	Temperature >38.5°C	Abdominal pain	ICU admission	Diabetes mellitus	Malign or hepatobiliary primary disease	Travel history^4^		Antibiotic resistance patterns^5^	String test result
Pat1	male	2022	54	blood, liver biopsy, BAL	yes	yes B, P, L	yes	yes	yes	diabetes mellitus type 1	no	no	TDM, CT drainage	no	positive
Pat2	male	2022	76	liver puncture, blood	yes	yes B, L	no	no	no	diabetes mellitus type 3c	pancreas carcinoma	no	CT drainage	no	negative
Pat3	female	2022	90	liver puncture	no	no	no	yes	no	no	cholecystitis	yes	CT drainage	no	positive
Pat4	female	2022	26	bronchoscopy, liver biopsy	no	yes P, L	yes	yes	no	no	no	yes	CT drainage	no	positive
Pat5	male	2022	no data	blood, biliary puncture	yes	yes, B, L	no	yes	no	no	cholecystitis	no	surgical	no	positive
Pat6	male	2023	55	liver drainage	no	no	no	no	no	no	no	yes	CT drainage	no	negative
Pat7	male	2023	81	liver puncture	yes	yes B, L	no	no	yes	no	CCC	no	CT drainage	no	negative
Pat8	male	2023	45	blood	yes	yes B, L	no	no	no	IDDM2	cholangitis, bile duct stenosis	no	No data	MDR	negative
Pat9	male	2023	50	blood, liver secretion	yes	yes, B, L	yes	no	yes	diabetes mellitus	no	yes	surgical	no	positive
Pat10	male	2023	61	blood	yes	yes B, L	yes	yes	yes	NIDDM2	no	no	CT drainage	no	positive
Pat11	male	2023	84	liver puncture, blood	yes	yes B, L	yes	yes	no	no	no	yes	CT	no	positive
Pat12	male	2023	45	liver puncture	no	no	no	yes	no	no	cholecystitis	no	CT	no	positive

^a^maximal diameter of the abscess; ^2^ all materials/specimen from which *K. pneumoniae* were isolated; ^3^ Location of disseminated Disease: B = bloodstream infection, *p* = pulmonary focus, L = liver abscess; ^4^ outside of Germany and relevant for infectious diseases; ^5^ solely remarkable multidrug resistance patterns were included; abbreviations: CT- Computed tomography, IDDM2- insulin dependent diabetes mellitus type 2, NIDDM2 - non insulin dependent diabetes mellitus type 2, TDM - therapeutic drug monitoring, BAL - bronchoalveolar lavage, ICU - intensive care unit.

WGS-based analyses confirmed that all isolates were *K. pneumoniae* with an average core gene identity of 99.8% and an average GC content of 57.3% (see supplemental table S1 for details). The paired-end sequencing generated 401,489–3,547,074 read pairs per isolate (mean sequencing depth 59x (range 22–198×), genome size 5.69–5.76 Mb). *De novo* assemblies (SPAdes) showed N50 values of 80–170 kb, L50 11–18, and BUSCO completeness of 100% (S: 96.5–99.8%, D: 0.2–3.5% using proteobacteria_odb10). The *de novo* reconstructed sequences provided insights into the genetic diversity (see supplemental table S2 for details) and phylogenetic relatedness (Figure 2).

As shown in Figure 2, the isolates were genetically divers, resulting in seven different sequence types (ST) and seven different *Klebsiella* capsule types (KL). Frequent ST-capsule combinations were identified, including ST23-KL1 (*n* = 6), ST25-KL2 (*n* = 4), and ST86-KL2 (*n* = 3). Isolates from the same patient (see Table 1) clustered closely together and were highly identical with 0-to-14 single nucleotide polymorphisms (SNPs) (see supplemental table S3). Only two isolates of Pat2 shared identical cgMLST types (see supplemental tables S2). The phylogenetic and typing results were consistent with the clinical survey, confirming that no transmission events were recorded. The general sequence type-based grouping in the phylogenetic tree showed 57-to-103 core SNPs within one ST-cluster and about 8,600-to-10500 core SNPs between different STs (see supplemental table S3).

Analysis of the sequences for antibiotic resistance genes revealed that all isolates except one (SPK41444) were classified as completely sensitive to all antibiotics except ampicillin (see supplemental table S4). Several resistance genes were identified in the genome of SPK41444, including the genes for an OXA-1 beta-lactamase and a CTX-M-15 ESBL. Phenotypic susceptibility testing confirmed resistance against three substance classes including piperacillin-tazobactam and ampicillin-sulbactam. The isolates SPK0391-1 and SPK0391-2 were also resistant to piperacillin-tazobactam and ampicillin-sulbactam, but no resistance determinants were identified. Therefore, SPK41444 was the only isolate classified as multidrug resistant (MDR; see Table 1).

In terms of virulence gene content and virulence score (VS), the collection included one isolate without virulence-associated genes (VS = 0) and three isolates carrying only yersiniabactin (VS = 1). The predominant lineages, ST23-KL1, ST86-KL2 and ST25-KL2, exhibited three to seven virulence-associated genes, with a VS ranging from 3 to 5. All of these isolates carried the *K. pneumoniae* virulence plasmid KpVP-1. SPK77933 was the only isolate with a VS of 4, and the only non-ST23 isolates that carried both KpVP-1 and an ICEKp. A total of five different types of ICEs were identified. In SPK0697, the virulence genes appeared to be chromosomally located, only ICEKp1 and a Col440II plasmid were identified. Primarily, IncHI1B (*n* = 13) was predicted, followed by IncFIB (*n* = 5). The plasmids could not be fully reconstructed from the short-read data.

### Patient cases collective

A total of 12 patients with monomicrobial *K. pneumoniae* LA were identified, of which 20 *K. pneumoniae* isolates were obtained. All infections were acquired on an outpatient basis; no nosocomial infections or transmissions were reported. The following table (Table 1) summarizes the clinical symptoms, patient history, and metadata. Of the 12 patients, 10 were male and 2 were female. The patients’ ages ranged from 41 to 88 y, with a median age of 64.5 y. Among the patients, eight male patients developed bacteremia, and two patients had additionally disseminated pulmonary infections. A total of five patients, all male, had a form of diabetes mellitus (DM). In addition, one patient had a malignant disease and four patients had liver and biliary tract diseases. Two patients (Pat7 and Pat8) died during their hospital stay in a causal or temporal relationship with their *K. pneumoniae* findings.

### Alignment of clinical information and genomic data

**Figure 3.**
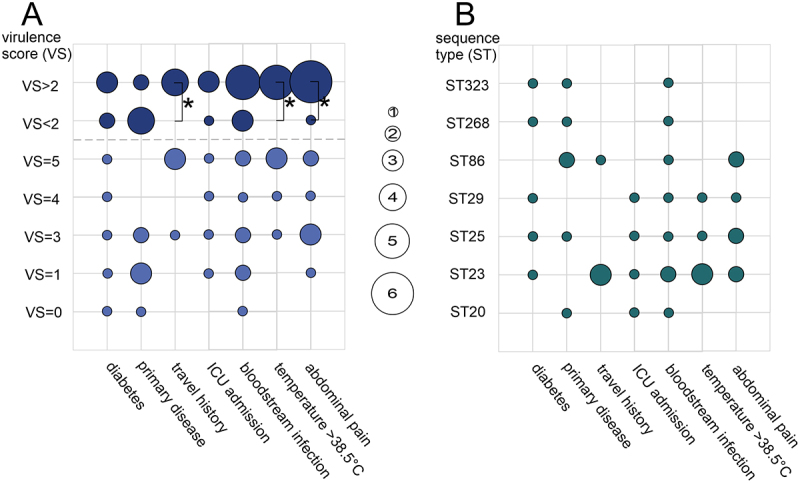
Alignment of genomic features and clinical investigations. The figure plots the frequency with which different A) virulence scores (vs) and B) sequence types (ST) were associated with the clinical records within the patient cohort. The observed frequencies are visualized as circles of varying sizes (size = number of patients). Only one isolate per patient was considered, even if multiple isolates were available. A) significant differences between groups VS > 2 and VS < 2 were highlighted (*).

The clinical information and genomic data were compared and matched. No correlation was found between abscess diameter and bacterial typing results, including sequence type and virulence gene content. Virulence scores and sequence types were compared with clinical observations linked to hvKp infections and are shown in Figure 3.

Figure 3 aims to highlight possible associations between bacterial typing and clinical presentations. In sum, virulence scores (VS) above “2,” appeared more often with clinical observations (Figure 3A). It was observed that VS = 5 was more often associated with travel history, abdominal pain and high temperature. Less clear correlations were observed for primary disease, ICU admission and bloodstream infection. Clustering the data by VS > 2 or VS < 2 revealed the significant association of abdominal pain (*p* = 0.033) and temperature >38.5°C (*p* = 0.033) with VS > 2. Also travel history was significantly (*p* = 0.048) correlated with VS > 2. Bloodstream infection, ICU admission and diabetes showed no significant (*p* = 1.000) correlations with VS but was more frequent in VS > 2. Interestingly, VS < 2 was in absolute numbers more common with primary diseases, such as underling malignancies or hepatobiliary diseases, but not statistically significant (*p* = 0.071).

With regard to sequence typing (ST; Figure 3B), all STs were found in bloodstream infection. The diagram provided a more detailed insight into the relationships between VS and clinical presentation. The correlations for specific clinical presentations were less clear compared to VS. The STs associated with VS > 2 allowed for differentiation and showed that ST23 had the most clinical correlations. Interestingly, ST86 was the only ST with VS > 2 that frequently occurred together with primary diseases. ST23 was the only ST identified in all three clinical presentations except primary disease.

**Figure 4.**
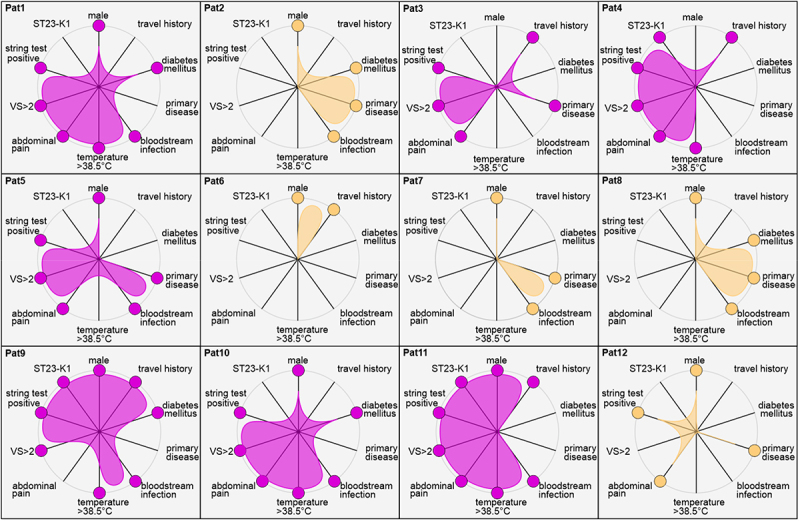
Overview of all included cases. The figure presents key characteristics of the 12 patients, including their underlying conditions, clinical presentations, and features of the corresponding *K. pneumoniae* isolates (see legend). No sequencing-based data were available for Pat6. The data is presented in a binary scheme, where a colored dot represents an observed characteristic. The filled areas and colors visualize the case interpretation: hvKpla = violet; KpLA = beige.

In addition, the study aimed to characterize and compare the condition of patients, clinical presentations, and characteristics of isolates, incorporating data from Table 1 and genome analyses. Based on these data, we sought to provide an overview of the individual clinical profiles (Figure 4).

As shown, the case presentations varied greatly, ranging from two-to-nine positive parameters. The case definition of hvKp was derived based on these clinical profiles. With a focus on molecular characterization, isolates carrying the KpVP-1 plasmid-coded virulence markers: aerobactin, salmochelin, *peg-344*, and *rmpA2* and/or *rmpADC* were considered as hvKp LA (hvKpLA): Pat1, Pat3, Pat4, Pat5, Pat9, Pat10, and Pat11. This classification is further supported by the presence of bloodstream infection (BSI), travel history, or diabetes mellitus as known risk factors and typical clinical presentations. Due to the number of cases, these observations could not be confirmed as statically significant. However, abdominal pain (*p* = 0.033) and fever (*p* = 0.008) were significant for hvKpLA cases according to two-sided Fisher’s exact test.

In contrast, Pat2, Pat7 and Pat8 are classified as *K. pneumoniae* LA (KpLA) due to the absence of hvKp-related genes. The case of Pat6 cannot be clearly defined due to a lack of molecular investigations, but based on the clinical presentation we define it as putative KpLA. Pat12 could potentially be considered as hvKpLA due to the presence of three virulence genes, but based on the clinical presentation, we also classify this case as KpLA.

Based on these classifications, the study identified 7/12 (58.3%) cases of hvKpLA and 5/12 (41.7%) cases of KpLA. Based on these results, the proportion of hvKpLA was estimated at 23.3% (7/30) and that of KpLA at 16.7% (5/30) of all *K. pneumoniae* cases in LA. Using these case definitions, case fatality rates (CFR) were calculated. The CFR for the KpLA was 40%, while the CFR for the hvKpLA group was 0%. In a two-sided exact Fisher’s test, there was no significant (*p* = 0.118) difference when comparing the CFR of both groups.

## Discussion

This study provides insights into the epidemiology of hypervirulent *K. pneumoniae* liver abscesses by combining clinical case observations with molecular investigations. We identified seven hvKpLA and five KpLA cases. HvKp was most commonly associated with ST23-KL1, ST86-KL2 genetic lineages. We have shown that the identification of hvKp in clinical diagnostics requires the integration of consistent microbiological biomarkers alongside the clinical manifestation of infection. Diabetes mellitus is suspected as potential risk factor for severe *K. pneumoniae* infections. The focus on monomicrobial LA should strengthen the study to avoid pathogenic effects of other bacterial species, as in polymicrobial infections.

Several decades ago, *E. coli* was identified as the most common bacterial pathogen in LA patients [12]. However, with the emergence of hypervirulent *K. pneumoniae*, the incidence of *K. pneumoniae* in LA increased, initially in Asia and now worldwide [50].

In our study, *K. pneumoniae* was identified in 27% of all LA cases, compared to 39% for *E. coli*. These observations differ from studies from China, which reported a higher prevalence of *K. pneumoniae* in LA [51,52]. However, when considering monomicrobial infections, our data are consistent with these studies, which identify *K. pneumoniae* as the most common pathogen in LA. In addition, the demographic data analyzed in these studies showed a comparable age and gender distributions to those observed in our cohort [51,52].

We calculated the frequency of hvKp in our study and estimated the prevalence of hvKp LA (hvKpLA) to be approximately 6.3%. This percentage is comparable to previous European studies; for example a French study found a value of 8.9% for hvKpLA [11]. The prevalence in Europe is lower than the 16.7% reported for hvKpLA in a Chinese study from 2016 [53]. A study from Taiwan suggested the differing prevalences in East Asia of LA might be directly correlated with higher numbers of hvKp [54]. In recent years, studies have indicated a global increase in LA numbers, which in turn appears to be linked to a general increase in *K. pneumoniae* and hvKp numbers [55]. However, it is difficult to compare these results on a global scale, as the frequency of hvKp varies between countries (12–45%) and depends on the specific definition of hvKp, especially since older studies have equated hypermucoviscosity and hypervirulence [56,57].

The question of defining hypervirulence becomes even more complex because clinical presentations are often more variable than the genotyping of hvKp itself, with manifestations extending beyond LA [19]. The case definitions used in this study are consistent with current recommendations in the literature [58], which commonly advocate a combination of genetic investigations with clinical presentations or experimental infection assays [57,59]. Therefore, we included widely used marker genes, including those used in the Kleborate virulence score (VS > 2), as well as the *rmpA* genes [60,61]. The virulence score alone is not as discriminating as incorporating all virulence-associated genes of the hypervirulence plasmid [61]. Choosing for VS > 2 as threshold was based on ensuring the presence of aerobactin, which is repeatedly reported as one major genetic marker for hvKp [57,60]. Regarding the importance for awareness in routine diagnostics, a positive string-test combined with VS > 2 could be considered as possible hvKp isolates for further WGS-based investigations. The hvKpLA definition should include clinical manifestations, together with genotyping, especially when known sequence types were identified by subsequent WGS analyses [37]. Notably, all isolates classified as hvKp cases in our study were also string-test positive, most likely due to carriage of one or more *rmpA* genes, while most non-hvKp cases were string-test negative. The string-test is rightly being discussed because of its limited specificity, but phenotypic assays for detecting hypervirulence features are valuable for comprehensive diagnostic surveillance [62]. This applies in particular when *K. pneumoniae* is isolated from significant clinical samples such as LA material or blood cultures [63,64].

Alongside with other investigations, the study intended to survey diabetes mellitus in hvKp cases. Several patients were diagnosed with a form of DM, but the statistical investigations and interdisciplinary hvKp case definitions led to inconclusive results. In general, systematic investigations of the relationship between diabetes and hvKp infections have only been possible to a limited extent to date, but indicated statistical correlations [65]. This is reasoned primarily due to the small number of confirmed hvKp cases on the one hand and the well-established association between *K. pneumoniae* urinary tract infections and DM patients on the other hand [66]. In addition, the estimated average age for DM diagnosis is around 61 y, which is roughly equivalent to the median age of 69 y observed in our patient cohort [67]. Therefore, a random-like distribution cannot be ruled out, and in our study, the number of cases was too small for valid statistical differentiation. It remains difficult to conclusively determine whether DM, and/or the level of metabolic control, is a reliable risk factor for hvKpLA [52]. Future studies need to use uniform case definitions, as well as defined patient collectives to clarify the associations of DM, hvKp and LA.

The statistically significant differences observed between hvKpLA and KpLA with regard to abdominal pain and fever (temperature > 38.5°C) are of limited medical significance and could be used as soft criteria in future studies. Further, the observed hints for a correlation of VS < 2 with primary diseases, as underling malignancies or hepatobiliary diseases, were not used for case definitions. This could be consistent with statements in the literature that define hvKp in otherwise healthy patients, but further studies with a higher number of cases are needed [68].

The genetic diversity observed in the isolates was unexpected, as one of our previous studies highlighted the dominance of the ST23/KL1 lineage [25]. Furthermore, we identified lineages that differ from those commonly found in Germany, as previously studied [37]. This suggests that invasive *K. pneumoniae* infections are driven by a diverse collection of genetic lineages with a variety of non-hvKp and non-cKp strains. On the other hand, we classified hvKpLA based on their association with globally prevalent hvKp lineages, such as ST23-KL1 and ST86-KL2, which all carry genes associated with hypervirulence. Interestingly, one isolate was identified as MDR, belonging to ST323. This genotype was highlighted in European hospitals as an emerging high-risk clone, particularly in connection with the KPC-2 spread [69]. The isolate in our study did not express carbapenemases but could be classified as convergent strain due to its MDR phenotype [70].

Spread beyond LA was observed for both hvKpLA and KpLA cases. In-patient spread was observed as bloodstream infection, and in two cases, as pulmonary infection. This observation was not statistically significant for the differentiation between hvKpLA and KpLA. With regard to hvKp isolates from different materials from a single patient, we were able to include blood culture isolates from some patients. These isolates were identical in terms of hypervirulence-associated genes and genotype, supporting the assumption of hvKp dissemination during infection and potential systemic spread [19].

The definition of hvKpLA from KpLA could not show a significant different CFR in our study. Mortality rates between 4% and 10% have been reported in the literature for hvKpLA, which is not comparable to the estimated mortality rates for invasive MDR *K. pneumoniae* (up to 60%) [54,71,72]. The evaluation of these results remains difficult, due to different study protocols, inclusion criteria and definitions. The higher CFR of KpLA cases in our study could also be related to the underlying malignant diseases of Pat7 and Pat8, as has been shown previously for *K. pneumoniae* bloodstream infections [73].

On the other hand, phylogenetic analyses showed no clonality between patients’ isolates, concluding no evidence for intra-hospital dissemination. Although the isolates belong to globally widespread hvKp lineage genotypes, we were unable to observe any clonal relationships or epidemiological correlations that would indicate transmission [48]. Despite studies from China and Japan report nosocomial acquisition of hvKp in 60–90% of investigated patient groups, our study setting eventuated differently [74,75]. The circulation of hvKp clones is evident, intra-hospital transmission and nosocomial infections appear to be unusual in Germany [37].

Our study had limitations. First, we focused only on the *K. pneumoniae* isolates from monomicrobial LA diagnosis. This excluded the polymicrobial infections and should be considered for comparative reasons in future. The restriction on monomicrobial cases was chosen to strengthen the study results in terms of direct correlation solely to hvKp, even knowing that it limits the case numbers and statistical power. Due to the study design, the final number of cases included did not allow for significant statistical results beyond gender distribution. Given the limited sample size, statistical analyses were exploratory, and results should be interpreted with caution and considered primarily hypothesis-generating for follow-up studies. Looking ahead, future studies should further investigate multidrug resistance patterns, as well as a more diverse clinical presentation beyond LA, e.g. bloodstream infections in general, as these were not part of our study cohort. Also, future studies could focus on *in vivo* infection models to affirm the hypervirulent pathotypes.

## Conclusion

The presented study provided a comprehensive overview of *K. pneumoniae* and hvKp in the context of LA. The in-depth case investigations, combined with genomics-based typing, allowed for a distinction between hvKp and non-hvKp cases. Furthermore, it proposed a reliable framework for differentiation, addressing the urgent need for uniform definitions and enhanced surveillance of clinical presentations. Future studies can build solidly on the data and experience gained, for example to implement diagnostic investigation of hvKpLA at national level. This perspective would offer deeper understanding of these severe infections and enable the exploration of possible regional differences.

## Supplementary Material

Supplement Data_rev.xlsx

## Access to data

All data generated or analyzed during this study are included in this published article and its supplementary information files. The article follows the Open Data policy of the journal. Raw sequencing data were submitted to NCBI-SRA under BioProject accession PRJNA1001661 (https://www.ncbi.nlm.nih.gov/bioproject/?term=PRJNA1001661), BioSample accessions SAMN48516985 - SAMN48517002.
